# Adaptation on a genomic scale

**DOI:** 10.7554/eLife.06193

**Published:** 2015-02-03

**Authors:** István Bartha, Jacques Fellay

**Affiliations:** School of Life Sciences, École Polytechnique Fédérale de Lausanne, Lausanne, Switzerland and Host-pathogen genomics group, Swiss Institute of Bioinformatics, Lausanne, Switzerland; School of Life Sciences, École Polytechnique Fédérale de Lausanne, Lausanne, Switzerland and Host-pathogen genomics group, Swiss Institute of Bioinformatics, Lausanne, Switzerland, jacques.fellay@epfl.ch

**Keywords:** Candida albicans, drug resistance, microbial evolution, genomics, virulence, other

## Abstract

Sequencing the genome of *Candida albicans* as it evolves in a patient reveals the genetic changes that allow the yeast to adapt to its environment.

**Related research article** Ford CB, Funt JM, Abbay D, Issi L, Guiducci C, Martinez DA, Delorey T, Li BY, White TC, Cuomo C, Rao RP, Berman J, Thompson DA and Regev A. 2015. The evolution of drug resistance in clinical isolates of *Candida albicans*. *eLife* **4**:e00662 doi: 10.7554/eLife.00662

**Image** *Candida albicans* cells taken from a patient before treatment with an antifungal drug (left), and after adapting to resist the drug (right)

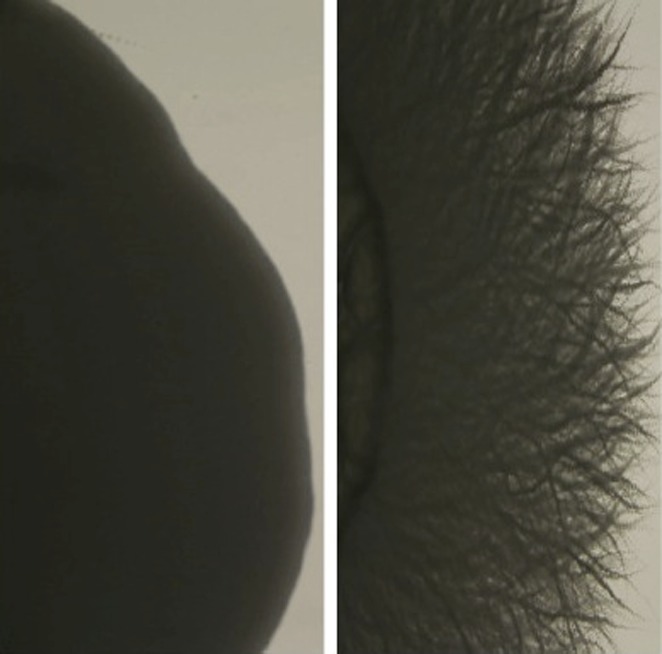

Adaptation to new environments is of fundamental importance in ecology. Infectious agents and cancer cells also relentlessly adapt to escape detection by the immune system and to avoid being killed by drugs targeted at them. The nature and extent of adaptive changes in various human pathogens have been studied under well-controlled conditions in the laboratory (Foll et al., 2014; Szamecz et al., 2014), and also in clinical samples (Mukherjee et al., 2011; Poon et al., 2012). Several mutations leading to drug resistance in *Candida albicans,* a species of yeast that causes oral and genital infections in humans, have been identified through targeted gene sequencing or large-scale genotyping approaches (MacCallum et al., 2010; Dhamgaye et al., 2012). Now, in *eLife*, Dawn Thompson and Aviv Regev of the Broad Institute of MIT and Harvard and co-workers—including Christopher Ford and Jason Funt as joint first authors—describe the genomic adaptation of *C. albicans* when it is exposed to a common antifungal compound (Ford et al., 2015).

*C. albicans* is found in a large fraction of the human population. Normally, it does not affect its host, but it can become pathogenic in people with weakened immune systems (Mayer et al., 2013). *C. albicans* has a diploid genome—a common feature of sexually reproducing species, featuring two sets of each chromosome—but has a predominantly asexual life cycle (Diogo et al., 2009). These genetic features are very similar to those of cancer cells (Landau et al., 2013).

To shed light on the development of drug resistant strains in vivo, Ford, Funt et al. studied samples from HIV-infected individuals diagnosed with a fungal infection called oral candidiasis and treated with fluconazole. This drug works by preventing *C. albicans* making a molecule called ergosterol that is incorporated into the cell membrane: without this molecule the yeast cell can't grow.

Ford, Funt et al. analyzed 43 fungal isolates collected from 11 individuals, with several samples taken from each individual over a period of several months. Using deep-sequencing technology, they obtained a whole genome sequence of *C. albicans* from each sample, which allowed them to produce a comprehensive catalogue of the different genetic variants of the fungus. These variants range from single nucleotide mutations in the DNA of the cells to large-scale genetic changes such as loss of heterozygosity (where one of the two copies of a chromosomal region is lost) and aneuploidies (where the cell contains either more or fewer chromosomes than normal). Of note, all sequencing data have been made publicly available, which is an unprecedented resource for the research community.

The first isolate, collected before treatment started, provided a snapshot of pre-existing genetic variation and was used to filter out non-drug-related mutations. To identify the genes that are under selection pressure during fluconazole therapy, Ford, Funt et al. searched for mutations called ‘non-synonymous coding single nucleotide polymorphisms’ that emerged and persisted in at least three of the patients under treatment. These mutations alter a single DNA nucleotide, which subsequently changes the identity of an amino acid in one of the proteins produced by the cell. Such mutations were observed in a total of 240 genes, notably including genes encoding proteins involved in fungal cell wall formation or in the regulation of the efflux pumps that move toxic substances out of the cell.

Among the larger-scale genetic variants, loss of heterozygosity events were significantly associated with higher drug resistance, whereas most aneuploidies were transient and had no detectable impact. However, aneuploidies may still indirectly help resistance to develop by increasing the likelihood that loss of heterozygosity events occur following the loss of a chromosome. Measurement of the in vitro fitness of the sequenced strains convincingly demonstrated that *C. albicans* had adapted to fluconazole: the measured fitness of the last isolate was indeed higher in the presence of the drug than without it.

Because a single colony was sequenced at each time point, it was not possible to distinguish between the appearance of new mutations and the selection of pre-existing minority variants in response to drug pressure. Most isolates from each individual were highly related, suggesting that samples collected in the same patient share the same common ancestor. Despite this clonal relationship, significant within-host diversity was present, as various isolates of the same patient differed by thousands of single nucleotide polymorphisms.

A remaining open question is whether pre-existing genetic variation is maintained at low levels in the *C. albicans* population throughout the treatment period, or if only drug-selected strains are conserved. To address this, more in-depth genomic analyses of fungal sub-populations are needed. An obvious first step would be to study the loss of heterozygosity events in greater detail, because such mutations are irreversible in clonal populations with a purely asexual life cycle. Therefore, valuable insights into the diversity of *C. albicans* inside a single host could be gained by investigating whether any of the resistance-inducing loss of heterozygosity events reverses after fluconazole treatment has ended.

The work by Ford, Funt et al*.* provides a global description of the genetic processes underlying drug resistance and adaptation in *C. albicans*. What else could now be learned about microbial evolution using deep sequencing technology? First, the analysis of multiple strains collected simultaneously in the same infected patient has the potential to reveal population structure, dynamics and diversity. Second, mechanistic and temporal details governing the emergence of escape mutations will certainly be gained from in vitro experiments, including the characterization of single colonies over time. Finally, the sequencing of paired host and pathogen genomes opens the door to innovative studies of host-specific adaptation.
